# Subsidies from anthropogenic resources alter diet, activity, and ranging behavior of an apex predator (*Canis lupus*)

**DOI:** 10.1038/s41598-019-49879-3

**Published:** 2019-09-17

**Authors:** Tyler R. Petroelje, Jerrold L. Belant, Dean E. Beyer, Nathan J. Svoboda

**Affiliations:** 1 Camp Fire Program in Wildlife Conservation, State University of New York, College of Environmental Science and Forestry, 1 Forestry Drive, Syracuse, New York 13210 USA; 2 Wildlife Division, Michigan Department of Natural Resources, 1990 US Highway 41 S, Marquette, MI 49855 USA; 3 Alaska Department of Fish and Game, 351 Research Court, Kodiak, AK 99615 USA

**Keywords:** Behavioural ecology, Conservation biology

## Abstract

Acquisition of resources can be costly and individuals are predicted to optimize foraging strategies to maximize net energy gain. Wolves (*Canis lupus*) would be expected to scavenge on subsidies from anthropogenic resources when these resources provide an energetic benefit over the capture of wild prey. We examined the effects of subsidies from anthropogenic resources in the form of livestock carcass dumps (LCDs) on wolf space use, activity, tortuosity, and diet in portions of North America’s northern hardwood/boreal ecosystem. We fitted 19 wolves with global positioning system collars during May–August of 2009–2011 and 2013–2015. Wolves with LCDs within their home ranges used areas adjacent to LCDs greater than non-LCD sites and had decreased home ranges and activity as compared to wolves without LCDs in their home ranges. Additionally, cattle comprised at least 22% of wolf diet from scavenging in areas with LCDs present as compared to no cattle in the diet of wolves without access to LCDs. Subsidies from anthropogenic resources in the form of LCDs can serve as attractants for wolves and alter wolf diet, activity, and ranging behavior. Apex predators may alter their behavior where subsidies from anthropogenic resources occur and management of these subsidies should be considered when attempting to reduce the impacts of humans on wolf behavior.

## Introduction

An optimal foraging strategy is one that maximizes net gain of energy^1,2^ and may be determined by prey distributions^3^. A generalist foraging strategy may be advantageous when prey resource availability changes seasonally and individuals exhibit prey-switching behavior to more readily available or energetically advantageous prey^4^. Large or consistent food resource subsidies, like those introduced by humans (e.g., bait piles, bird feeders, agricultural fields), occur rarely or intermittently naturally^5^, but can result in switching to these food resources^6,7^. Stable, high-quality food resources like these would therefore be preferred, particularly if they provide an energetic advantage over irregular or dispersed food resources^8^.

Space use, and more specifically home range size, is correlated with metabolic demand and distribution of resources consumed^9,10^. Some species that are central place foragers continually return to one site (i.e., den site, nest cavity, etc.) following foraging bouts that radiate from those sites^11^. However, when resources are highly abundant, shifts in space use may occur resulting in smaller than expected home ranges predicted from energetic requirements^12^. Facultative scavenging too may occur when a stable influx of resources such as subsidies from anthropogenic resources (hereafter called food subsidies) becomes available^6^; use of these food subsidies may alter individuals’ diet, space use, and social structure^7^. Use of these food subsidies, particularly by apex predators, may also result in some level of risk due to human-caused mortality^13^. Behavioral changes have been observed across a variety of taxa associated with various food subsidies such as fishing discards (black-legged kittiwake [*Rissa tridactyla*]^14^), landfills (silver gull [*Larus novaehollandiae*]^15^; coyote [*Canis latrans*]^16^), supplemental feeding (black-capped chickadee [*Poecile atricapilla*]^17^), and refuse in urban areas or human settlements (red fox [*Vulpes vulpes*]^18^; raccoon [*Procyon lotor*]^19^; dingo [*Canis lupus dingo*]^20^).

Wolves (*Canis lupus*) are opportunistic central place foragers during the spring and summer when pup rearing^21^. However, due to the greater metabolic demand of wolves because of their body size, pack behavior, and specialized diet they tend to have large home ranges relative to other North American canids^9^. Though wolves are often viewed as a symbol of wilderness^22^, on average 32% of their diet has been attributed to food subsidies worldwide^23^. Wolves in North America use food subsidies such as carrion from hunter killed moose (*Alces alces*)^24^, livestock carcass dumps (LCDs) on rangeland^25^, and appear to visit LCDs with varying frequency in the upper Great Lakes region^26^.

Wolves prey largely on ungulates but seasonal dietary shifts occur in response to prey vulnerability. Reduced vulnerability of ungulates to wolf predation during summer, often a period of nutritional stress for wolves, results in a more generalist diet^27^. Although wolf response to LCDs has been found to increase during the non-grazing season for free ranging cattle^25^, in the Great Lakes Region it is not clear when wolves visit LCDs^26^. During the summer period of lesser prey vulnerability and greater nutritional demand during pup rearing, LCDs may be an important resource for wolves and thus may influence their diet and ranging behavior.

We studied indices of wolf diet, activity, and ranging behavior in areas with and without known food subsidies from LCDs. Here, we hypothesized that wolf diet, activity, and ranging behavior follow the expectations from foraging theory within the constraints of a central place forager during early pup rearing and use resources that provide increased calories with decreased energy expenditure. We tested our hypothesis by identifying wolf home range and core use areas, activity, active step tortuosity, proportion of cattle (*Bos tarus*) in diet, and site use around known LCDs compared to wolves in areas without access to LCDs. We predicted wolves exposed to food subsidies would exhibit a reduced home and core range, greater use of LCD areas relative to availability, and lesser activity and straighter active trajectories, and include these subsidies in their diet due to increased food availability and reduced energy expenditure searching for and acquiring food.

## Study Area

**Figure 1:**
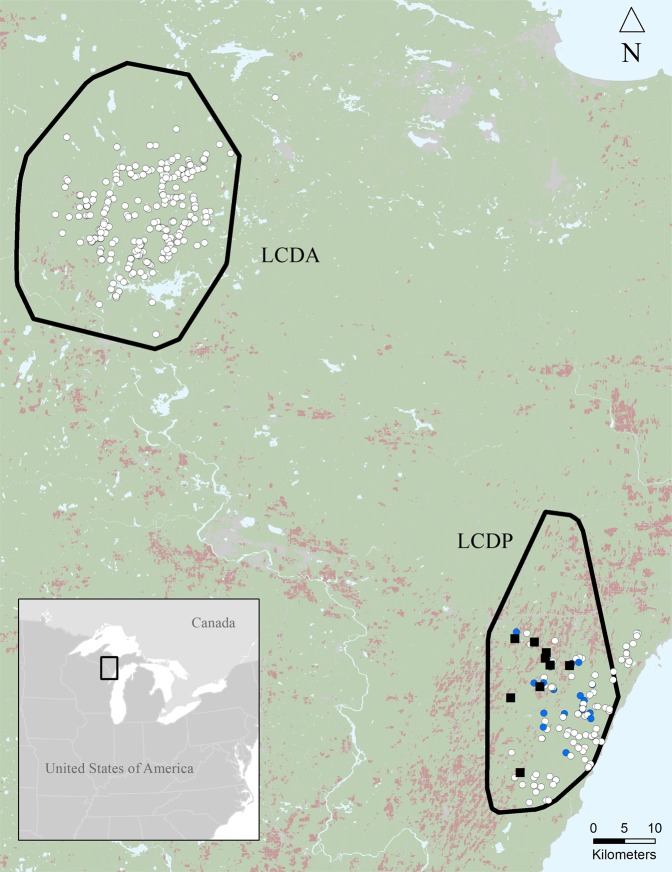
Minimum convex polygons calculated from all GPS locations of resident wolves (black polygons) in areas with presence of known livestock carcass dumps (■; LCDP) and areas absent of known livestock carcass dumps (LCDA). Locations of collected wolf scat with (blue circle) and without (white circle) presence of cattle in scat remains. Distribution of agriculture (i.e., row crops and pastures [brown]), developed (light grey), other land cover types (e.g., forested, wetlands [green]), and water (light blue) identified from 2011 National Land Cover Database (Jin *et al*. 2013). Inset showing the location of the study region (black rectangle) within North America. Michigan’s Upper Peninsula, USA (46.0 Latitude, −87.7 Longitude), 2009–2011, 2013–2015.

We conducted this study in two areas separated by about 80 km (Fig. 1) in the Upper Peninsula of Michigan, USA (46.0 Latitude, −87.7 Longitude). The first area, with LCDs present (LCDP), was mostly forested with woody wetlands and deciduous hardwoods (77%) and interspersed with agriculture (18%; row crop, hay field, and livestock dairies) across the landscape^28^ (Fig. 1). Wolf density in the LCDP area, estimated by winter track surveys (Supplementary Methods), was 1.4 individuals/100km^2^ during 2009–2011 with a mean pack size of 5.5 (Supplementary Table S1). Human population density was 8.9/km^2^ within Menominee County^29^ where most wolf home ranges occurred. Road density within the LCDP area wolf home ranges was 1.03 km/km^2^. Elevations ranged from 177 to 296 m. The area without LCDs (LCDA) was mostly forested (86%) with little agriculture (<1%; potato farms, no livestock operations) and dominant land cover types included deciduous hardwood forests and woody wetlands^28^ (Fig. 1). Wolf density in the LCDA area, estimated by winter track surveys, was 2.8 individuals/100km^2^ during 2013–2015 with a mean pack size of 5.6^30^. Human population density was 3.9/km^2^ within Iron County^29^ were most wolf home ranges occurred. Road density (0.48 km/km^2^) within wolf home ranges in the LCDA area was lesser than the LCDP area. Elevations ranged from 401 to 550 m.

## Results

### Wolf capture and home ranges

**Figure 2:**
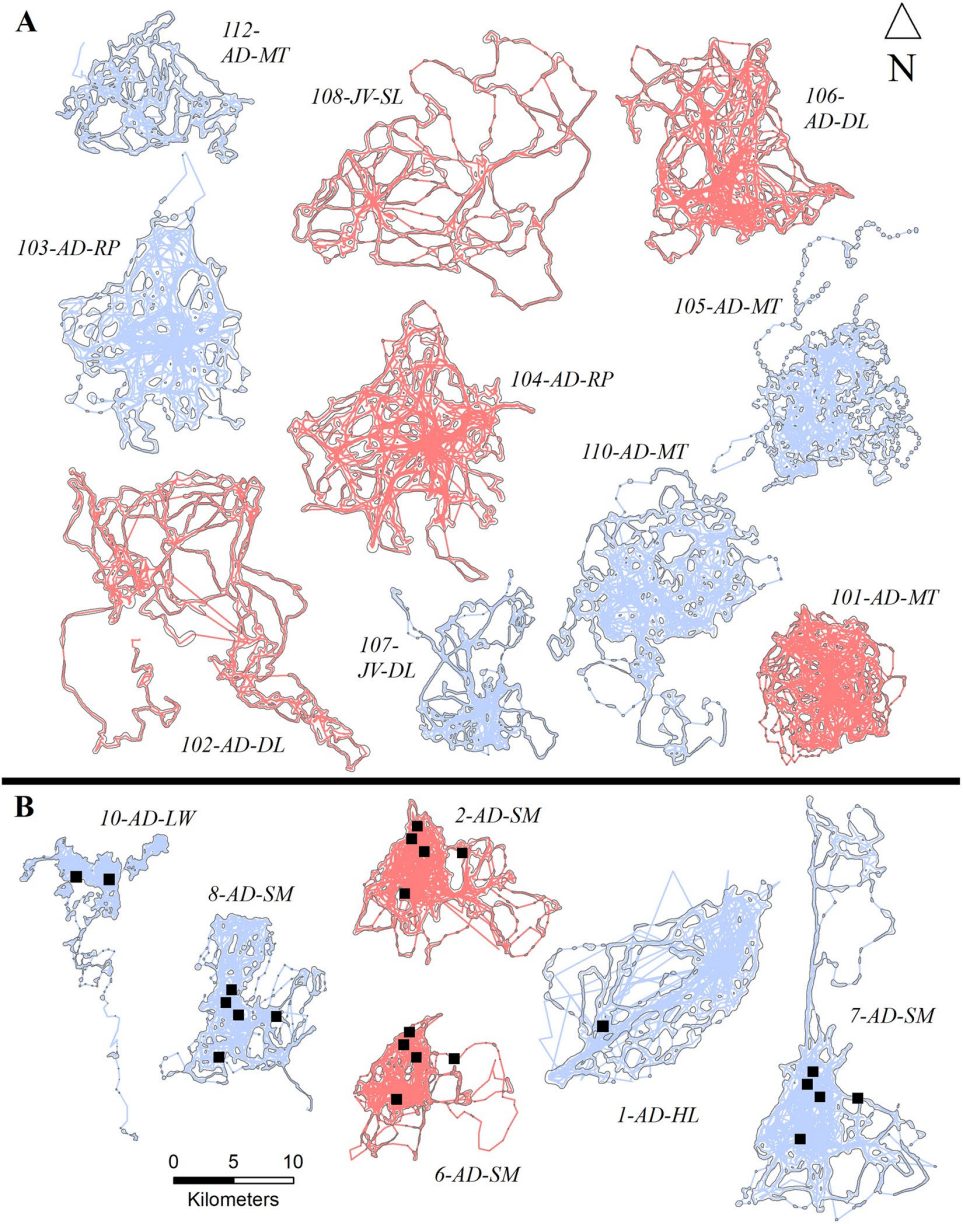
Wolf home ranges estimated with dynamic Brownian Bridge movement models (99% utilization distribution; gray line) and GPS line movements (red = male, blue = female) in areas with (B) and without (A) livestock carcass dumps (LCDs). Home ranges and movements are displayed to be non-overlapping however the scale is the same across individuals. Each wolf home range is labeled with wolf identification number, age (AD = adult, JV = juvenile), and pack (DL = Deer Lake, HL = Hayward Lake, LW = Lone wolf, MT = Mitchigan, RP = Republic, SL = Shank Lake, and SM = 7-mile marsh). Additionally, when applicable, locations of LCDs (■) are noted within each wolf home range. Michigan’s Upper Peninsula, USA, 2009–2011 and 2013–2015.

We captured and collared 8 wolves (2 male, 6 female) from 3 packs within the LCDP area during May–June 2009–2011, and 11 wolves (5 male, 6 female) from 4 packs within the LCDA area during May–June 2013–2015. We used collar data collected during May–August for analysis of each study area and excluded three wolves from analysis: one female from each area due to dispersal and one female we considered transient (home range = 688.08 km^2^) crossing multiple pack territories within the LCDP area. Wolves in the LCDP area wore collars for 102–121 days ($\bar{x}$ = 116, SD = 6.6) and we collected 9,792–11,615 locations per individual ($\bar{x}$ = 11,136; SD = 636.8). Wolves in the LCDA area wore collars for 25–119 days ($\bar{x}$ = 93, SD = 24) and we collected 2,400–11,424 locations per individual ($\bar{x}$ = 8,944, SD = 2,317). No individual wolves wore collars for more than one consecutive year. We only knew of the breeding status of one adult female wolf (wolf 103; Fig. 2) which was lactating at the time of capture in the LCDA area. We were unable to determine the breeding status of all other captured wolves and thus report only age, sex, and pack in Fig. 2. Resident wolf core areas were similar (*P* = 0.757, t = 0.72, df = 8.5) in the LCDP area (0.09–0.58 km^2^) and the LCDA area (0.05–0.38 km^2^). However, resident wolf home ranges in the LCDP area (15.23–46.97 km^2^) were 1.87 times smaller on average (*P* = 0.004, t = -3.05, df = 13.8) than home ranges in the LCDA area (20.10–85.09 km^2^; Table 1).

**Table 1:** Parameter estimates and test statistics for multiple comparisons between wolves with livestock carcass dumps present (LCDP, *n* = 6), and wolves with livestock carcass dumps absent (LCDA, *n* = 10) from their home ranges. Michigan’s Upper Peninsula, USA, 2009–2011, 2013–2015.

Parameter	LCDP		LCDA		Test statistic _*df*_	*P*-value
	Estimate	SD	Estimate	SD		
Home range^a^	31.14	14.03	58.34	21.60	t = −3.05 _*13.8*_	0.004
Core range^a^	0.28	0.18	0.22	0.14	t = 0.72 _*8.5*_	0.757
50 m use [LCD]^b^	0.45	0.04	—	—	z = 10.56 _354_	<0.001
200 m use [LCD]^b^	0.58	0.02	—	—	z = 28.51 _386_	<0.001
Activity^c^	42.26	1.71	49.61	10.57	t = −1.90 _*7.9*_	0.047
Tortuosity^d^	66.77	3.79	60.01	3.67	t = 2.74 _*3.4*_	0.969
Diet^e^	0.22	0.37	0.00	—	—	—

^a^Area (km^2^) calculated with 99% (Home range) or 50% (Core range) utilization distribution estimated by dynamic Brownian Bridge movement models.

^b^Generalized linear mixed model estimates of use within 50 m and 200 m of livestock carcass dumps [LCD] clusters as compared to non-LCD clusters.

^c^Mean activity level estimated from collar mounted accelerometer readings.

^d^Mean relative angles between steps during daily activity bouts (greater value indicates a more tortuous movement).

^e^Proportion of wolf scat identified as cattle by volume.

### Livestock carcass dump site use

We investigated 256 clusters in the LCDP area and identified 9 LCDs. Of the 256 clusters investigated, 13% were at a LCD (Table 2). All but one LCD was associated with a dairy farm and near live cattle. In the LCDA area, we investigated 538 clusters and did not identify any LCDs. Collared wolves in the LCDP area exhibited greater site use within 50 and 200 m of a LCD cluster compared to non-LCD clusters within their home range (Table 1).

**Table 2:** Site-use determination of investigated wolf global positioning system (GPS) clusters with 5 or more GPS locations in regions with (*n* = 256) and without (*n* = 538) livestock carcass dumps (LCD). Michigan’s Upper Peninsula, USA, 2009–2011, 2013–2015.

Site-use determination	^a^LCD present	^a^LCD absent
Denning	2.5	1.5
Predation/scavenging	9.5	17.8
Livestock carcass dump	13.0	0.0
Undetermined use	32.0	42.8
Bedding/rendezvous site	43.0	37.9

^a^Percentage of sites investigated; may not reflect true percent of occurrence as we reduced visitation to, or avoided, certain sites once identified (i.e., den sites, livestock carcass dumps) and only visited locations where wolves spent ≥ 1 hour.

### Activity and tortuosity

Of the 16 resident collared wolves in the LCDP and the LCDA areas, we recovered activity data for 14 wolves (*n* = 4, LCDP; *n* = 10, LCDA). Activity for wolves in the LCDA area was 1.24 times greater than for wolves in the LCDP area (*P* = 0.047, t = −1.90, df = 7.9; Table 1). Turning angles along active paths traveled by wolves were not less tortuous in the LCDP area than the LCDA area (*P* = 0.969, t = 2.74, df = 3.4).

### Diet

We collected and analyzed 480 wolf scat samples identified by the presence of wolf tracks nearby (*n* = 151) or their diameter (*n* = 329) from the LCDP (*n* = 152) and LCDA (*n* = 328) areas for identification of percent by volume of prey remains in scats. Wolf scats found in the LCDP area contained 70% white-tailed deer (62% adult, 8% fawn; *Odocoileus virginianus*), 22% cattle, and 6% eastern cottontail rabbit (*Sylvilagus floridanus*) or snowshoe hare (*Lepus americanus*). Wolf scats found in the LCDA area contained 78% white-tailed deer (40% adult, 38% fawn), 0% cattle, 3% eastern cottontail rabbit or snowshoe hare, and 19% Rodentia. In the Michigan Department of Natural Resources wolf depredation database, we found 1 record of a livestock depredation event that occurred within a collared adult female wolf home range. The livestock depredation occurred on a dairy farm and involved 1 heifer in August 2010.

## Discussion

We identified four aspects of wolf diet or ranging behavior which we suggest were influenced by the presence of LCDs. Wolves with access to LCDs exhibited smaller home ranges, greater site use at LCDs than non-LCD clusters, and lesser activity than wolves without access to LCDs. Additionally, at least 22% of the wolf scats collected in areas where wolves had access to LCDs contained cattle demonstrating use of LCDs in the wolves’ diet.

The influence of LCDs on wolf ranging behavior was scale dependent. Wolves with access to LCDs had home ranges that were almost half the size of wolves without LCDs in their home ranges, but core areas of wolves did not differ in size. Being central place foragers, wolf core area size may be similar with or without access to LCDs because they focus activity around den and rendezvous sites during this time of the year^21^. The reduced home ranges of wolves with access to LCDs compared to wolves without suggests a difference in behavior linked to differences in resource availability^12^ which we attribute to LCD. This has been demonstrated by other canids like red fox^31^ and dingoes^32^ which exhibited smaller home range sizes when food subsidies were accessible. Further, red foxes more than doubled the size of their range following removal of food subsidies^31^.

It is also important to examine densities of wild prey as this could influence wolf home range size if they differ between study areas. White-tailed deer densities were greater in areas with LCDs (3.9–4.9 adult female deer/km^2^)^33^ than without LCDs (2.1–2.6 adult female deer/km^2^)^30^. However, proportion of deer found in wolf scats in the LCDP area comprised a relatively lesser portion of the wolf diet compared to wolf scats found in areas without LCDs. Additionally, wolves with access to LCDs contributed to a lesser proportion of fawn mortality than wolves in areas without food subsidies^30,34^. This suggests that home range size was more likely influenced by presence of LCDs than prey availability.

At least 13% of wolf clusters occurred at LCDs. This estimate is conservative as we could not visit all clusters created by the algorithm. Our estimates include investigated clusters and we did not always reinvestigate newly identified clusters from subsequent data downloads near known dens or LCD areas. Consistent with our predictions and at both spatial scales, wolves exhibited greater use of LCD clusters than non-LCD clusters, undoubtedly because of reliable and easily obtainable food. Food and water subsidies altered dingo space-use such that it was clumped near these resources^35^. Similarly, raccoon altered their distribution and space-use resulting in patchy use of home ranges where reliable food from food subsidies existed^19^. In addition to space-use, wolves with access to LCDs exhibited lesser activity suggesting reduced energy expenditure as compared to wolves without LCDs. Reductions in energy expenditure when food subsidies are available can result from less time searching for and acquiring dispersed prey as seen with dingoes near refuse sites^32^. These reductions in activity may be particularly pronounced during an energetically costly period like rearing young, as seen in nesting black-legged kittiwakes where supplemental feeding reduced energy expenditure by nearly one third^36^.

Interestingly, wolves with access to LCDs did not have less tortuous movements along active trajectories as compared to wolves without LCDs. We hypothesized greater straight-line travel could equate to less searching behavior which would be likely given a dependable resource such as a LCD. It is possible that path tortuosity does not accurately reflect searching behavior or habitat quality alone but also is in response to the landscape mosaic^37^. Wolves are more likely to use roads with low human activity and tortuosity of wolf movements is greater near roads or trails with high human activity^37–39^. The LCDP area contained a patchwork of agricultural fields, primary paved roads, and greater human density whereas the LCDA area was dominated by contiguous forests, secondary roads, and lesser human density. Greater use of secondary roads for traveling and hunting in the LCDA area may explain the similarity of movement behavior with wolves in the LCDP area but warrants further analysis.

Due to the energetic benefits of food subsidies, positive numerical responses may be expected^8^. For example, Fedriani *et al*.^40^ observed coyotes in human-dominated landscapes subsidized by anthropogenic foods and found densities eight times greater than coyotes in areas feeding on natural foods. Similarly, greater dingo group sizes occurred in areas with access to food subsidies than in areas without^32^. Wolf densities and group sizes were not greater in the LCDP area compared to the LCDA area which may be due to differences in land-use practices by humans. In the LCDP area 18% of landcover was agriculture with greater human and road densities limiting the space that can be occupied by wolves compared to <1% of the LCDA area which was mostly forested and had a lesser human and road density. It is also possible that LCDs do not provide food resources in excess of what would be needed to experience a numerical response considering LCDs appear to provide less than half of the May–August diet.

Ecosystem processes are often altered when apex predators rely on food subsidies, and their functional ecological roles can be altered, especially in areas where humans cause high mortality rates of carnivores due to conflict^8,41^. The possible effects of wolf use of LCDs on ecosystem processes are not well known. Our findings suggest that wolves obtained at least 22% of their diet from LCDs during May–August, often a period of nutritional stress^27^. Without these food subsidies, at least 22% of the diet of wolves would need to be acquired from other prey sources such as white-tailed deer, lagomorphs, rodents, or potentially livestock. Additionally, the reduced size of home ranges and activity by wolves in the LCDP area may allow conspecific carnivores to exploit other prey resources not being used by wolves. For example, coyotes in the LCDP area occur at high densities (0.32–0.37/km^2^)^42^ and are an important predator of white-tailed deer neonates during summer^34^. Thus, prey species populations such as white-tailed deer could experience overall increased predation pressure, with the dominant predator species changing seasonally.

Open pit LCDs are illegal in Michigan (Public Act 239 of 1982) and wolf behavior is affected by LCDs. Improved enforcement of livestock dumping laws would decrease livestock carcass availability and force wolves to change their foraging behaviors. For example, when a pulsed resource is depleted, facultative scavengers may exhibit prey-switching and increase predation on alternative prey^43,44^. In Oregon, wolves reduced time spent in a region with known LCD’s from 58% to 7% following hazing and removal of LCD’s and switched to preying on an elk (*Cervus elaphus*) resource (R. Brown, personal communication, 20 October 2017). Alternatively, closure of LCDs could result in an increased risk of depredations on live cattle due to habituation where LCDs previously existed as seen in spotted hyenas (*Crocuta crocuta*), where when food subsidies were scarce, they increased predation on domestic donkeys^45^. Although we only monitored LCDs for 3 consecutive years, many appeared to have been established at least several years prior and though we have not quantified the density of, or biomass available at, LCDs in Michigan’s Upper Peninsula, this practice is common among livestock owners in Michigan (B. Roell, personal communication, 04 November 2017), Minnesota^26^, as well as on livestock rangelands in Alberta^25^. Thus, food subsidies likely supplement the diet of wolves and alter the behavior of this apex predator where wolf range overlaps with livestock operations.

The ecosystem effects of removing LCDs are undescribed but when food subsidies are present, the outcomes can be negative for wildlife species, ecosystem functions, and humans^7,8^. However, the closure and removal of other food subsidies suggests some species become dependent once habituated to a stable subsidy. Following the removal of dumps in Yellowstone National Park, brown bears (*Ursus arctos*) body size and reproductive success decreased, and home range and human-bear conflict increased resulting in a decline of the brown bear population^46^. Similarly, vulture populations that fed on livestock carcasses stagnated, with declines in breeding success and increased mortality of young following legislation requiring sanitary disposal of livestock carcasses across southern Europe^47^. We demonstrated that LCDs can alter the diet and ranging behavior of wolves and suggest the need to monitor impacts of food subsidies on apex predators in other areas where subsidies are available^48^. Additionally, as other studies have pointed out the need to monitor responses to the removal of these subsidies is equally as important to better understand the effects of subsidies on apex predators^7,8,13^. As human activities increasingly alter landscapes, effective management of resulting food subsidies should be considered when attempting to reduce human effects on apex predator behavior.

## Methods

### Wolf capture

We captured wolves during May 2009–2011 in the LCDP area and May–June 2013–2015 in the LCDA area. We used foothold traps (model MB-750; Minnesota Trapline Inc., Pennock, Minnesota, USA) set along roads to capture wolves and checked traps at least once daily. We immobilized captured wolves with an intramuscular injection of ketamine hydrochloride (10 mg/kg; Ketaset^®^, Fort Dodge Laboratories, Inc., Fort Dodge, IA) and xylazine hydrochloride (2 mg/kg; X-Ject E^TM^, Butler Schein Animal Health, Dublin, OH)^49^. We administered yohimbine hydrochloride (0.15 mg/kg; Hospira^©^, Forest Lake, IL) to reverse the effects of xylazine hydrochloride before we released wolves at the capture site. Before release, we applied an ear tag with a unique identifier to each ear and affixed a Lotek 7000SU Global Positioning System (GPS) radio-collar (Lotek Wireless Inc., New Market, Ontario, Canada) with an on-board tri-axial accelerometer to record wolf locations and activity, respectively. We programmed collars to obtain a GPS location every 15 minutes from 1 May to 31 August of each year (2009–2011 and 2013–2015) and to record and store accelerometer data averaged over 5 minute periods for the x (side-to-side) and y (front-to-back) axis. Collars were equipped with a timed released drop-off mechanism for recovery in September–October of each year. We uploaded collar data to a handheld device approximately once weekly while flying via airplane. We received approval for all capturing and handling procedures through Mississippi State University’s Institutional Animal Care and Use Committee (protocol 09-004 and 12-012). Additionally, all wildlife handling methods were carried out in accordance with State of Michigan regulations.

### Identification of livestock carcass dumps

We used GPS locations of collared wolves to identify potential predation sites (clusters). We developed an algorithm in program R (v. 3.0.0, R Foundation for Statistical Computing, Vienna, Austria; http://www.r-project.org) which identified clusters as ≥8 (during 2009–2011) or ≥4 (during 2013–2015) 15-minute locations occurring within a 50-m radius within a 24 hour period (available in the GitHub repository [https://github.com/tpetroel/GPS_Cluster_Code]). Two investigators, with or without detection dogs, visited clusters and searched a 50-m radius to search for signs of prey remains^50,51^ and classified sites as denning, predation/scavenging, LCD, undetermined use, or resting/rendezvous site based on available evidence.

### Scat collection

We collected wolf scats opportunistically while traveling along roadways or conducting other field work throughout both areas during May–August of 2009–2011 (LCDP) and 2013–2015 (LCDA). We labeled collected scats with date of collection, location, presence of wolf tracks in the substrate next to the scats, and diameter of scats, then froze samples until further analysis.

### Wolf home ranges

We estimated home ranges (99% utilization distribution [UD]) and core areas (50% UD) of resident collared wolves using dynamic Brownian Bridge Movement Models (dBBMM) provided in package move (v. 3.0.2) and calculated area for each UD with package adehabitatHR (v. 0.4.0) available for program R. We set the dBBMM window to 25 locations, margin to 11 locations, time step to 1-min, and raster size to 30 × 30 m. Additionally, we included an error vector for estimated error at each GPS location defined by number of satellites used, dilution of precision provided by GPS collars. We determined an individual to be resident if it was associated with other collared wolves^52^ or displayed a maintained home range during May–August, returning following extraterritorial movements. We assessed if wolf home ranges and core areas encompassed less area in the LCDP area compared to the LCDA area using a Welch two sample t-test $({\mu }_{LCDP}-{\mu }_{LCDA} < 0)$.

### Livestock carcass dump use

We created buffers of 50 and 200 m around each known LCD cluster and known non-LCD cluster we visited in the LCDP area. We then calculated number of GPS locations within each buffer from the wolf used to create each cluster. We excluded all clusters within 100 meters of known denning sites biased from disproportionately greater use during pup rearing (T. Petroelje unpublished data). We chose these distances to be representative of areas searched around potential wolf predation sites (50 m)^51^ and areas where wolves may disproportionately use predation sites (200 m)^53^. We used a generalized linear mixed model with response as number of locations within each buffer to assess the influence of LCDs on wolf use compared to other clusters. We included cluster type (i.e., LCD or non-LCD) as the explanatory variable and wolf ID as a random effect.

### Activity and tortuosity

We used accelerometer data from wolf collars as a representative measure of activity. We calculated mean activity of wolves from the sum of the x and y axis accelerometer values for all 5-minute intervals. We estimated if mean activity for wolves was lesser in the LCDP area compared to wolves in the LCDA area using a Welch two sample t-test. We used the package adehabitatLT (v. 0.3.2) in program R to calculate mean turning angles (i.e., difference in angle between two consecutive steps; also called the relative angle^54^) along active trajectories of wolves as a measure of search effort along a travel path. We considered wolves to be active if the mean activity for each location along the trajectory was greater than 30.8 (unitless measure) based on activity data paired with observations of captive collared wolves (T. Petroelje, unpublished data). We assessed if mean tortuosity of active turning angles of wolves was lesser in the LCDP area as compared to wolves in the LCDA area using a Welch two sample t-test.

### Diet

When available, we used diameter of scats with associated wolf tracks ($\bar{x}$ = 33.3 mm, SD = 6.1 mm, *n* = 151) to determine size diameter limits for scats without associated tracks to differentiate wolf scats from sympatric canids (i.e., coyotes). We considered scats without tracks that were <29 mm (first quantile of wolf scats) to be unknown as these overlapped with coyote scats ($\bar{x}$ = 25.1 mm, SD = 4.4 mm, *n* = 204) above the third quantile (28.2 mm); greater than the <24 mm cut-off recommended by Thompson^55^. We wrapped scats in double wrapped nylons and washed in warm water to remove all material except hair, vegetation, and bone fragments^42^. We estimated the percent by volume within each scat containing cattle, eastern cottontail and snowshoe hare, adult and fawn white-tailed deer, and prey remains of Rodentia using light microscopy of scale and banding patterns, coloration, and length of hair to determine species and age class for white-tailed deer^56–59^. Criticisms of using volume of prey items in scat is that large prey items tend to be under represented and small prey items overrepresented due to differences in surface ratios of indigestible matter^60^. We considered this in our interpretation of the results and note that our estimates of adult deer and cattle in wolves’ diets are conservative and therefore not used to estimate caloric intake. We did not identify trace (≤1%) prey items and ignored vegetation and soil in diet estimates as we were primarily interested in consumption of major prey items and cattle. To identify if any proportion of consumed cattle were attributed to livestock depredation, we examined depredation records from the Michigan Department of Natural Resources wolf depredation database (D. Beyer, unpublished data) that occurred within the 100% minimum convex polygons of resident wolf GPS locations during 2009–2011 and 2013–2015 (Fig. 1).

## Supplementary information

Supplementary Information

## Data Availability

All data collected and analyzed during this study are available from the corresponding author on reasonable request.

## References

[1] MacAurthur RH, Pianka ER (1966). On optimal use of a patchy environment. American Naturalist.

[2] Perry G, Pianka ER (1997). ‘Animal foraging: Past, present and future’. Trends in Ecology and Evolution.

[3] Iwasa Y, Higashi M, Yamamura N (1981). Prey distribution as a factor determining the choice of optimal foraging strategy. American Naturalist.

[4] Murdoch WW (1969). Switching in general predators: experiments on predator specificity and stability of prey populations. Ecological monographs.

[5] Yang LH, Bastow JL, Spence KO, Wright AN (2008). What can we learn from resource pulses?. Ecology.

[6] Dijk JV (2008). Diet shift of a facultative scavenger, the wolverine, following recolonization of wolves. Journal of Animal Ecology.

[7] Oro D, Genovart M, Tavecchia G, Fowler MS, Martínez-Abraín A (2013). Ecological and evolutionary implications of food subsidies. Ecology Letters.

[8] Newsome TM (2015). The ecological effects of providing resource subsidies to predators. Global Ecology and Biogeography.

[9] Paquet, P. C. & Carbyn, L. N. Gray wolf in *wild mammals of North America* (eds Feldhamer, G. A., Thompson, B. C. & Chapman, J. A.) 482–510 (Johns Hopkins, 2003).

[10] Macdonald D (1983). The ecology of carnivore social behavior. Nature.

[11] Rosenberg DK, McKelvey KS (1999). Estimation of habitat selection for central-place foraging animals. Journal of Wildlife Management.

[12] McNab BK (1963). Bioenergetics and the determination of home range size. American Naturalist.

[13] Newsome TM, Van Eeden LM (2017). The effects of food waste on wildlife and humans. Sustainability.

[14] Regehr HM, Montevecchi WA (1997). Interactive effects of food shortage and predation on breeding failure of black-legged kittiwakes: indirect effects of fisheries activities and implications for indicator species. Marine Ecology Progress Series.

[15] Smith GC, Carlile N (1993). Food and feeding ecology of breeding silver gulls (*Larus novaehollandiae*) in urban Australia. Colonial Waterbirds.

[16] Hidalgo-Mihart MG, Cantu-Salaar L, Lopez-Gonzalez CA, Fernandez EC, Gonzalez-Romero A (2004). Effect of a landfill on the home range and group size of coyotes (*Canis latrans*) in a tropical deciduous forest. Journal of Zoology.

[17] Wilson WH (2001). The effects of supplemental feeding on wintering black capped-chickadees (*Poecile atricapilla*) in central Maine: population and individual responses. Wilson bulletin.

[18] Contesse P, Hegglin D, Gloor S, Bontadina F, Deplazes P (2003). The diet of urban foxes (Vulpes vulpes) and the availability of the anthropogenic food in the city of Zurich, Switzerland. Mammalian Biology.

[19] Prange S, Gehrt SD, Wiggers EP (2004). Influences of anthropogenic resources on raccoon (*Procyon lotor*) movements and spatial distribution. Journal of Mammalogy.

[20] Newsome TM (2014). Human-resource subsidies alter the dietary preferences of a mammalian top-predator. Oecologia.

[21] Packard, J. M. Wolf behavior: reproductive, social, and intelligent in *Wolves: Behavior, Ecology, and Conservation* (eds Mech L. D. & Boitani L.) 35–65 (University of Chicago Press, 2003).

[22] Newsome TM (2017). Making a new dog?. BioScience.

[23] Newsome TM (2016). Food habits of the world’s grey wolves. Mammal Review.

[24] Lafferty DJR, Loman ZG, White KS, Morzillo AT, Belant JL (2016). Moose (*Alces alces*) hunters subsidize the scavenger community in Alaska. Polar Biology.

[25] Morehouse AT, Boyce MS (2011). From venison to beef: seasonal changes in wolf diet composition in a livestock grazing landscape. Frontiers in Ecology and the Environment.

[26] Mech LD, Harper EK, Meier TJ, Paul WJ (2000). Assessing factors that may predispose Minnesota farms to wolf depredations on cattle. Wildlife Society Bulletin.

[27] Peterson, R. O. & Ciucci P. The wolf as a carnivore in *Wolves: Behavior, Ecology, and Conservati*on (eds Mech L. D. & Boitani L.) 104–130 (University of Chicago Press, 2003).

[28] Jin S (2013). A comprehensive change detection method for updating the National Land Cover Database to circa 2011. Remote Sensing of Environment.

[29] United States Census Bureau. *QuickFacts*, https://www.census.gov/en.html (2010).

[30] Kautz, T. M. *et al*. Predator densities and white-tailed deer fawn survival in a four-predator system. *Journal of Wildlife Management* in press (2019).

[31] Bino G (2010). Abrupt spatial and numerical responses of overabundant foxes to a reduction in anthropogenic resources. Journal of Applied Ecology.

[32] Newsome TM, Ballard G, Dickman CR, Fleming PJS, van de Ven R (2013). Home range, activity and sociality of a top-predator, the dingo: a test of the Resource Dispersion Hypothesis. Ecography.

[33] Duquette JF, Belant JL, Svoboda NJ, Beyer DE, Albright CA (2014). Comparison of occupancy modeling and radiotelemetry to estimate ungulate population dynamics. Population Ecology.

[34] Duquette JF, Belant JL, Svoboda NJ, Beyer DE, Lederle PE (2014). Effects of maternal nutrition, resource use and multi-predator risk on neonatal white-tailed deer survival. PLoS ONE.

[35] Newsome TM, Ballard G, Dickman CR, Fleming PJS, Howden C (2013). Anthropogenic resource subsidies determine space use by Australian arid zone dingoes: an improved resource selection modelling approach. PLoS ONE.

[36] Jodice PGR (2002). Does food availability affect energy expenditure rates of nesting seabirds? A supplemental-feeding experiment with Black-leeged Kittiwakes (*Rissa tridactyla*). Zoology.

[37] Whittington J, St. Clair CC, Mercer G (2005). Spatial responses of wolves to roads and trails in mountain valleys. Ecological Applications.

[38] Thurber JM, Peterson RO, Drummer TD, Thomasma SA (1994). Gray wolf response to refuge boundaries and roads in Alaska. Wildlife Society Bulletin.

[39] James ARC, Stuart-Smith AK (2000). Distribution of caribou and wolves in relation to linear corridors. Journal of Wildlife Management.

[40] Fedriani JM, Fuller TK, Sauvajot RM (2001). Does availability of anthropogenic food enhance densities of omnivorous mammals? An example with coyotes in southern California. Ecography.

[41] Ordiz A, Bischof R, Swenson JE (2013). Saving large carnivores, but losing the apex predator?. Biological Conservation.

[42] Petroelje TR, Belant JL, Beyer DE, Wang G, Leopold BD (2014). Population-level response of coyotes to a pulsed resource event. Population Ecology.

[43] Ostfeld RS, Keesing F (2000). Pulsed resources and community dynamics of consumers in terrestrial ecosystems. Trends in Ecology and Evolution.

[44] Wilmers CC, Stahler DR, Crabtree RL, Smith DW, Getz WM (2003). Resource dispersion and consumer dominance: scavenging at wolf- and hunter-killed carcasses in greater Yellowstone, USA. Ecology Letters.

[45] Yirga G (2012). Adaptability of large carnivores to changing anthropogenic food sources: Diet change of spotted hyena (*Crocuta crocuta*) during Christian fasting period in northern Ethiopia. Animal Ecology.

[46] Despain, D., Houston, D., Meagher, M. & Schullery, P. Wildlife in transition: man and nature on Yellowstone’s northern range 1–142 (Roberts Rienehart, 1986).

[47] Donazar JA, Cortés-Avizanda A, Carrete M (2009). Dietary shifts in two vultures after the demise of supplementary feeding stations: consequences of the EU sanitary legislation. European Journal of Wildlife Research.

[48] Moss, W. E., Alldredge, M. W., Logan, K. A. & Pauli, J. N. Human expansion precipitates niche expansion for an opportunistic apex predator (*Puma concolor*). *Scientific Reports*10.1038/srep39639 (2016). 10.1038/srep39639 PMC5180354 28008961

[49] Kreeger, T. J. Handbook of wildlife chemical immobilization, third edition. 271 (Wildlife Pharmaceuticals, 2007).

[50] Paula J (2011). Dogs as a tool to improve bird-strike mortality estimates at wind farms. Journal for Nature Conservation.

[51] Svoboda NJ, Belant JL, Beyer DE, Duquette JF, Martin JA (2013). Identifying bobcat *Lynx rufus* sites using a global positioning system. Wildlife Biology.

[52] Hinton JW (2016). Space use and habitat selection by resident and transient red wolves (*Canis rufus*). PLoS ONE.

[53] Sand H, Zimmermann B, Wabakken P, Andrèn H, Pedersen HC (2005). Using GPS technology and GIS cluster analyses to estimate kill rates in wolf-ungulate ecosystems. Wildlife Society Bulletin.

[54] Calenge C, Dray S, Royer-Carenzi M (2009). The concept of animal’ trajectories from a data analysis perspective. Ecological Informatics.

[55] Thompson DQ (1952). Travel, range, and food habits of timber wolves in Wisconsin. Journal of Mammology.

[56] Mathiak HA (1938). A key to hairs of the mammals of southern Michigan. Journal of Wildlife Management.

[57] Adorjan, A. S. & Kolenosky, G. B. A manual for the identification of hairs of selected Ontario mammals 1–64 (Department of Lands and Forests, Ontario, Canada, 1969).

[58] Spiers, J. K. A microscopic key to the hairs of Virginia land mammals 1–96 (Virginia Polytechnic Institute and State University, 1973).

[59] Wallis RL (1993). A key for the identification of guard hairs of some Ontario mammals. Canadian Journal of Zoology.

[60] Floyd TJ, Mech LD, Jordan PA (1978). Relating wolf scat content to prey consumed. Journal of Wildlife Management.

