# Supplementary material for: Subsidies from anthropogenic resources alter diet, activity, and ranging behavior of an apex predator (Canis lupus)
Source: Sci Rep. 2019 Sep 17;9:13438. doi: 10.1038/s41598-019-49879-3 (PMC6748928; doi:10.1038/s41598-019-49879-3)
Supplement: Supplementary file 1 — Supplementary Information [file 41598_2019_49879_MOESM1_ESM.docx]

**Supplementary Information**

**Article in *Scientific Reports***

**Subsidies from anthropogenic resources alter diet, activity, and ranging behavior of an apex predator (*Canis lupus*)**

Tyler R. Petroelje, Jerrold L. Belant, Dean E. Beyer, Jr. & Nathan J. Svoboda

**Supplementary Methods**

We completed track surveys during December–March 2010 and 2011 generally within 12–24 h following snowfall and no later than 72 h to allow time for wolf movements and limit deterioration of tracks^1,2^. The use of winter snow track surveys to estimate abundance of many large mammal species is common^3,4^. Huntzinger et al.^5^ estimated abundance of wolves in Michigan’s western Upper Peninsula using the same track survey technique as did the Michigan Department of Natural Resources (MDNR) but with greater effort and reported similar abundance estimates (4% difference) of wolves as the MDNR survey. Consequently, we followed the winter track survey design of the MDNR wolf tracking surveys protocol^6^.

We traveled by truck, snowmobile, or ATV on established roads and trails at 8–24 km/hr to locate wolf tracks. We traversed >25% of drivable roads and trails^6^, with priority given to secondary roads, in areas of known wolf territories. When wolf tracks were located, we followed tracks if necessary, to estimate the number of individuals traveling together. We traveled roads multiple times until we were confident no more individuals were in the area^7^ as not all pack members always travel together^8^. To reduce chances of double counting individual tracks, observed tracks were circled on neighboring roadways following the direction of travel to observe if individuals remained between roads or if they have crossed to another area. Additionally, we used movement data from GPS collared wolves within the study area to estimate pack boundaries, assuming no newly formed packs, to avoid overestimating the number of packs. For each observation, we recorded date, time, location, suspected pack ID, and number of individuals. Additionally, we recorded time since last snowfall to estimate maximum track age and scent-marks such as urination, defecation, and scratching to aid in identifying territorial pairs and pack boundaries^6^.

References

1. Hayward, G. D., Miquelle, D. G., Smirnor, E. N., Nations, C. Monitoring Amur tiger populations: characteristics of track surveys in snow. Wildlife Society Bulletin **30**, 1150-1159 (2002).
2. Wydeven, A., Sabor, A. A., Schultz, R. N., Megown, R. A., Boles, S. R., Wiedenhoeft, J. E. Guidelines for carnivore tracking surveys during winter in Wisconsin. Wisconsin Department of Natural Resources, Madison, Wisconsin, USA (2004).
3. Becker, E. F., Spindler, M. A., Osborne, T. O. A population estimator based on network sampling of tracks in the snow. The Journal of Wildlife Management **62**, 968-977 (1998).
4. Patterson, B. R., Quinn, N. W. S., Becker, E. F., Meier, D. B. Estimating wolf densities in forested areas using network sampling of tracks in snow. Wildlife Society Bulletin **32**, 938-947 (2004).
5. Huntzinger, B. A., Vucetich, J. A., Drummer, T. D., Peterson, R. O. Wolf recovery in Michigan, 2002-05 Summary. Michigan Technological University, Houghton, Michigan (2005).
6. Michigan Department of Natural Resources and Environment Estimating wolf abundance in Michigan. East Lansing, Michigan, USA (2008).
7. Potvin, M. J., Drummer, T. D., Vucetich, J. A., Beyer, D. E., Jr., Peterson, R. O., Hammill, J. H. Monitoring and Habitat Analysis for Wolves in Upper Michigan. Journal of Wildlife Management **69**, 1660-1669 (2005).
8. Mech, L. D., Boitani, L. Wolf social ecology in *Wolves; behavior, ecology and conservation* (ed. Mech, L. D. and Boitani, L.) 1-34 (University of Chicago Press, 2003).

**Supplementary Table S1** Estimates of minimum number of wolves identified in each pack, mean pack size, and wolf density from winter wolf track surveys in areas with livestock carcass dumps, Michigan’s Upper Peninsula, USA, 2010–2011

| Year | Number of packs identified | Pack name | Pack size | Mean pack size (SD) | Estimated density (individuals/ 100 km^2^) |
| --- | --- | --- | --- | --- | --- |
| 2010 | 2 | 7-mile | 7 | 5.5 (2.1) | 1.38 |
|  |  | Hayward Lake | 4 |  |  |
| 2011 | 2 | 7-mile | 6 | 5.5 (0.7) | 1.38 |
|  |  | Hayward Lake | 5 |  |  |
